# Cross-Cultural Consumer Acceptability for Ethnic Fermented Sauce Products: Comparisons among Korean, UAE, and US Consumers

**DOI:** 10.3390/foods9101463

**Published:** 2020-10-14

**Authors:** Mi-Ran Kim, Seo-Jin Chung, Koushik Adhikari, HyeWon Shin, Hana Cho, Yerim Nam

**Affiliations:** 1 Department of Nutritional Science and Food Management, Ewha Womans University, Seoul 03760, Korea; ranni1027@kfri.re.kr; 2 Research Group of Food Processing, Korea Food Research Institute, Jeollabuk-do 55365, Korea; 3 Department of Food Science and Technology, The University of Georgia, Griffin, GA 30223, USA; koushik7@uga.edu; 4 CJ Cheiljedang, Inc., Seoul 04560, Korea; hyewon.shin@cj.net (H.S.); hn.cho1@cj.net (H.C.); yr.nam@cj.net (Y.N.)

**Keywords:** salad dressing, dipping sauce, *doenjang*, *gochujang*, global cuisine, drivers of liking

## Abstract

The present study investigated the drivers of liking ethnic sauces in a cross-cultural context. Experiments were conducted to understand the acceptance of salad dressings and dipping sauces developed from Korean fermented seasonings among consumers with different ethnic backgrounds, including: South Korean, United Arab Emirates (UAE), and American. The samples of interest included four types of salad dressings made from fermented soybean paste (*doenjang*) and four types of spicy dipping sauces made from fermented chili pepper paste (*gochujang*). The salad dressings were preferred by Korean and US consumers. Koreans liked the nutty-flavored salad dressings, whereas UAE and American consumers commonly liked the spicy type. There was a stronger cross-cultural agreement in liking dipping sauces rather than salad dressings. Both Korean and American consumers liked spicy dipping sauces that elicited a sweet taste. UAE consumers tended to prefer the less spicy dipping sauce samples. Consumers in all three countries generally liked spicy dipping sauces more than salad dressings. Cultural differences were observed between the responses depending on the presence and level of spiciness in the two different food types. For product development with ethnic fermented flavors or chili spices, the contextual appropriateness and consumer familiarity with the corresponding flavor should be taken into account.

## 1. Introduction

Ethnic foods are often referred to as traditional foods that are unique and familiar to consumers sharing a specific food culture, but are perceived as exotic or unfamiliar to others [1,2,3]. Particular food materials, spices, as well as authentic preparing and cooking processes uniquely developed within a culture, contribute to the ethnic identity of food [4]. In the past couple of decades, ethnic foods have gained considerable popularity among consumers around the world because of the increased accessibility and exposure to these foods through international trade, immigration, tourism, and media [5,6,7,8]. Along these trends, a so called “culinary tourism” has been promoted successfully in many countries [9,10,11]. Sauces, including condiments and dressings, are effective vehicles to characterize the cultural identity of a food. Thus, partly driven by the increased popularity of ethnic food consumption, the global market size of sauces was USD 136.24 billion in 2019, and the global compound annual growth rate of sauces is expected to steadily increase by 4.8% from 2015 to 2025 [12].

Along with these trends, the Korean food industry is also making efforts to globalize traditional Korean food items [13]. The major countries importing Korean foods are the US, Japan, and China. On the contrary, the Muslim population, although considered as an emerging global market with strong potential in the growth of halal food products, comprises a relatively small sector in the Korean food industry for food exports [14]. However, companies are seeking opportunities to expand their sales to Muslims. Fermented soy-based products such as soy sauce, fermented chili pepper paste (*gochujang*), and fermented soybean paste (*doenjang*) are essential seasoning materials to flavor and characterize traditional Korean cuisine [15,16]. One of these seasonings is almost always applied to flavor foods in Korean cooking. Among these seasonings, soy sauce has gained familiarity and popularity among consumers worldwide through the successful introduction of Chinese and Japanese cuisines in Western countries. Many studies have investigated the possible application and acceptability of soy sauce by consumers in foreign countries [17,18,19]. Compared with soy sauce, fermented chili pepper paste and fermented soybean paste are relatively new, and their usage is limited among non-Korean consumers.

Investigations of food acceptance and preference under a cross-cultural context have been pursued by many researchers with various aims and scopes during the past several decades [7,20,21,22]. Factors significantly influencing the cross-cultural variance in food preference and perception were analyzed. Overall, differences in cross-cultural acceptances are often reported when familiarities to the target food item or flavor are culturally different [7,23]. Cultural factors seemed to be more influential than genetic variability between subjects in the cross-cultural studies. Psychographic traits [24], consumption habits [25], and product information [26] are some of the other variables studied to understand the cultural differences in food acceptances. 

Recently, a check-all-that-apply question format (a.k.a. CATA method) is favorably exercised in the consumer sensory research area to gain insights on the consumer’s perception of a target food product [27]. The CATA method asks subjects to select all the relevant items from a preselected list of descriptions or terms. The flexible and versatile nature of the method has encouraged a wide usage in marketing for several decades [28]. Since Adams et al. [29] introduced the CATA application in the field of sensory science, the method has been commonly used to profile food products in terms of sensory or nonsensory (emotions, product usage, etc.) characteristics [7,27,30]. Providing a list of descriptions or terms to the subjects can motivate them to concentrate more on the product evaluation. Given that the terms are preselected, it is important to carefully select a pool of relevant attributes that subjects would consider during the product evaluation [31]. 

Many food cultures derive pleasure from salads and dips. Hence, in the present study, fermented soybean paste and spicy chili pepper paste were incorporated into salad dressing and dipping sauces, respectively, to examine the possibility of exporting these products to non-Asian countries. The sensory acceptability of *doenjang*-based salad dressings and *gochujang*-based spicy dipping sauces, both varying in flavor characteristics, were investigated among consumers in Korea, the United Arab Emirates (UAE), and the US. Korean consumers were selected as a control group. UAE and US consumers served as the target groups because of their precedence in the export of Korean foods. Additionally, the two countries were specifically chosen for their diverse culinary backgrounds, representing the food cultural groups of Middle Eastern Asia and North America, respectively.

## 2. Materials and Methods

### 2.1. Subjects

Consumer subjects who were interested in taste-testing Korean traditional fermented sauce products were recruited from the following three regions: Seoul (Korea), Al Ain (UAE), and Griffin (GA, US) and henceforth termed KOR, UAE, and US consumers, respectively. Consumers were informed that they would be evaluating various types of Korean fermented salad dressing and sauce products. The consumers were students and staffs at the local universities, as well as residents residing in each of the three regions. The experiments were carried out in the sensory labs of Ewha Womans University, the University of Georgia, and the University of Arab Emirates for Korea, UAE, and US regions, respectively. The study included 91 KOR consumers (aged 20–70 years), 60 US consumers (aged 18–68 years), and 70 UAE consumers (aged 18–63 years). The sex and ethnicity information of participating consumers are presented in Table 1. In this study, the experiments conducted in Korea and the UAE were approved by the Institutional Review Board (IRB) at Ewha Womans University, and those undertaken in the US were approved by the IRB at the University of Georgia. 

### 2.2. Food Samples

The products of interest included 4 types of salad dressings (based on *doenjang*, a Korean fermented soybean paste)—original, sour, nutty, and spicy—and 4 types of dipping sauces (based on *gochujang*, a Korean fermented chili pepper paste)—sweet (SW), sour (SO), spicy + sweet (SWSP), and spicy + sour (SOSP). The ingredients and manufacturer information on each product are listed in Table 2. 

### 2.3. Sample Preparation and Presentation

The samples were prepared based on the sample producer’s guidelines (CJ Cheiljedang, Seoul, Korea). Salad dressing samples were required to be diluted with water before serving. Thus, these were diluted with water at a salad dressing sample-to-water ratio of 8:2 (g/g) for presentation. For spicy dipping sauces, the samples were served as is without any additional preparation. Samples were prepared 24 h before the taste-test experiments. Thirty grams of sample was poured into a disposable container of 7 cm in diameter × 4 cm in height (Samboopack Corp., Incheon, Korea) for KOR and UAE consumers or 59-mL capacity (Dart Container Co., Ltd., Mason, MI, USA) for US consumers, and labeled with a random 3-digit code. All samples were stored in the refrigerator (4 °C) and removed from storage 1 h before testing.

Salad dressing and dipping sauce are rarely consumed on their own [3,8] and thus vegetables were served as carrier foods. Lettuce is the most typical vegetable used for making salads, although various vegetables can be used. Thus, romaine lettuce was selected as the carrier food for the salad dressing experiment. On the contrary, when considering dips for vegetable, a wide spectrum of vegetables with very different flavor characteristics can be candidates (carrots, cucumbers, broccoli, celery, etc.). It was shown in a preliminary test that consumers have extremely different preferences for different types of vegetables. Thus, for the dipping experiment, consumers were provided with vegetable options from which they can choose.

For the salad dressing experiment, the heart of the romaine lettuce was removed, and the leaves were cut to about 2–3 cm length. A total of 10 g of romaine lettuce was presented with a disposable fork. For the spicy dipping sauce, the vegetables of choice were English cucumber, baby carrot/carrot, and broccoli. The cucumber was cut into 4 halves horizontally and then divided again into 4 halves vertically. The seeds in the middle were removed. Baby carrots were provided in the US and UAE, while whole carrots were used in Korea. The whole carrot was cut to a similar size of the cucumber segments. The stem of the broccoli was removed, and the floret part was served. In total, 1 stick/flower of cucumber, carrot, and broccoli was provided per sample. The samples and carrier foods were provided separately so that the consumers could apply as much salad dressing or dip to the carrier foods as desired. 

### 2.4. Consumer Taste-Test Procedure

All consumers participated in two consecutive testing sessions, tasting fermented soybean paste-based salad dressing in the first session, and hot pepper paste-based dipping sauce in the second session. This order was chosen because the flavors of the salad dressing samples were considerably milder than those of the spicy dipping sauce samples. However, consumer subjects received the samples in balanced order using factorial design within the 4 salad dressing samples and within the 4 dipping sauce samples. All the KOR and US consumers completed both sessions, but 9 UAE consumers did not complete the evaluation of the dipping sauce samples. There was a 10 min break between the two sessions.

The overall testing procedures were modified from the methods described in the studies of Kim et al. [16] and Choi et al. [20]. When tasting each sample, consumers rated the overall, appearance, smell/odor, taste/flavor, and texture liking on a standard 9-point hedonic scale (1 = dislike extremely, 5 = neither like nor dislike, 9 = like extremely) [32]. Additionally, viscosity (both salad dressing and dipping sauce), saltiness (salad dressing only), and hot and spicy level (dipping sauce only) of each sample were evaluated on a 9-point just-about-right (JAR) scale. Consumers rated their familiarities with the flavors of the samples by checking the levels of agreement with the following statement: “I am familiar with the aroma and flavor of this salad dressing/dipping sauce.” The agreement scale was a 9-point category scale with word categories labeled from 1 = “not familiar at all” to 9 = “extremely familiar.” The reasons for (dis)liking each sample were surveyed using a check-all-that-apply (CATA) method. The CATA list of attributes comprised sensory characteristics and nonsensory characteristics (i.e., holistic and emotional terms). Warm water and unsalted crackers (Carr’s Original Table Water, Carr’s of Carlisle Ltd., Carlisle, UK) were provided to the consumers to cleanse the palate between samples.

### 2.5. Statistical Analysis

Concerning the acceptance testing data, analysis of variance (ANOVA) procedures were performed using the generalized linear mixed (GLM) model to determine the influences of the salad dressing/dipping sauce sample and the consumer’s country of origin on the acceptance. The GLM model used was salad dressing acceptance/intensity/familiarity = sample + country + (sample × country) + (country × panel), and dipping sauce acceptance/intensity/familiarity = sample + country + carrier food + (sample × country) + (sample × carrier food) + (country × carrier food) + (sample × country × carrier food) + (country × carrier food × panel). The effect of sample, country of origin, and carrier food were considered as fixed, while the consumer was designated as a random effect. Duncan’s multiple range test was used for post hoc comparisons of country or sample (*p* < 0.05). The one-sample *t*-test (two-tailed) was carried out on the intensity data obtained from the JAR scale. The intensity value was tested for its significant difference (at the 95% confidence interval) from the value “5”, which is the JAR midpoint [33]. Levene’s test was conducted to compare the equality of variances of the liking scores among the 3 countries. Pearson’s correlation analysis was conducted on the overall liking and familiarity ratings.

For CATA data, terms that were selected by more than 25% of the consumers were chosen for further analysis. Correspondence analysis (CA) was undertaken to visually summarize how consumers in the 3 countries perceived the samples. All statistical analyses were performed using IBM SPSS Statistics 21 (SPSS, Inc., Chicago, IL, USA), XLSTAT (Addinsoft, Paris, France), and EXCEL 2013 (Microsoft Corp., Redmond, WA, USA).

## 3. Results

### 3.1. Salad Dressing

#### 3.1.1. Effect of Product and Country of Origin on Consumers’ Acceptance and Perception Ratings

The influence of sample, country, and sample × country interaction effects on the ratings of liking, intensity, and familiarity of salad dressing samples analyzed by GLM are shown in Table 3. Additionally, Levene’s test showed that the equality of variances of the liking ratings differed among the three countries (*p* < 0.001).

**Figure 1:**
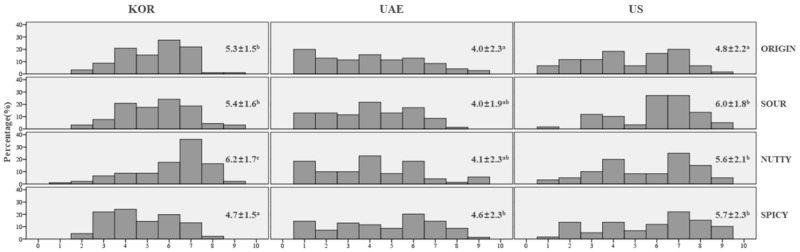
Histogram of overall liking ratings (mean ± SD) of salad dressing samples evaluated by KOR, UAE, and US consumers. KOR, UAE, US denote South Korean, United Arab Emirates, and Americans, respectively. ^‡^ Samples sharing the same superscript letters within the same country do not differ significantly among the products (α = 0.05).

The cross-cultural distribution of overall liking ratings, mean values, and standard deviations for the four salad dressing samples (Figure 1) showed that KOR and US consumers gave higher mean overall liking scores for the samples than the UAE consumers (mean scores, 5.4, 5.5, and 4.2 respectively). Each country exhibited a different preference order for the four salad dressing samples. KOR consumers rated overall liking for the nutty sample the highest (6.2), and the spicy sample the lowest (4.7). By contrast, UAE consumers assigned significantly higher overall liking scores to spicy (4.6) than other salad dressing samples but even this sample did not exceed “neither like nor dislike”, indicating that the UAE consumers did not like any of the samples. UAE consumers selected “dislike extremely” (16.5%) most frequently among all three populations. Most of the salad dressing samples were given a similar level of acceptance among the US consumers, with overall liking scores ranging between 5.7 to 6.0, except for the original sample, which was rated the lowest (4.8). As can be observed from the overall liking histogram, US consumers tended to avoid selecting the “neither like nor dislike” category in the hedonic scale, which resulted in a bipolar distribution. In comparison to the other samples, the spicy sample displayed a rather flat distribution of liking, revealing a weak consensus on the liking level among the consumers.

Cross-cultural differences were observed in the ratings of perceived salty and thickness intensities as well (Table 4). Saltiness perception of all four salad dressing samples was strongest among KOR consumers (mean saltiness intensity = 6.6), followed by US (5.8) and UAE consumers (5.0). However, consumers from all three countries agreed that the sour sample was the least viscous. Differences existed in both the flavor familiarity ratings by country and in the familiarity rating patterns by sample. Familiarity scores of KOR consumers (mean familiarity score = 5.3) were generally higher than those of the US (4.0) and UAE (3.4) consumers. KOR consumers perceived the nutty sample as being the most familiar (5.7). UAE consumers rated the familiarity of spicy and nutty samples higher than the other samples. US consumers did not perceive any significant difference in familiarity among the samples. The familiarity ratings showed a significant positive correlation with the overall liking scores by correlation analysis (0.35–0.38).

#### 3.1.2. Reasons for Liking and Disliking the Samples

**Figure 2:**
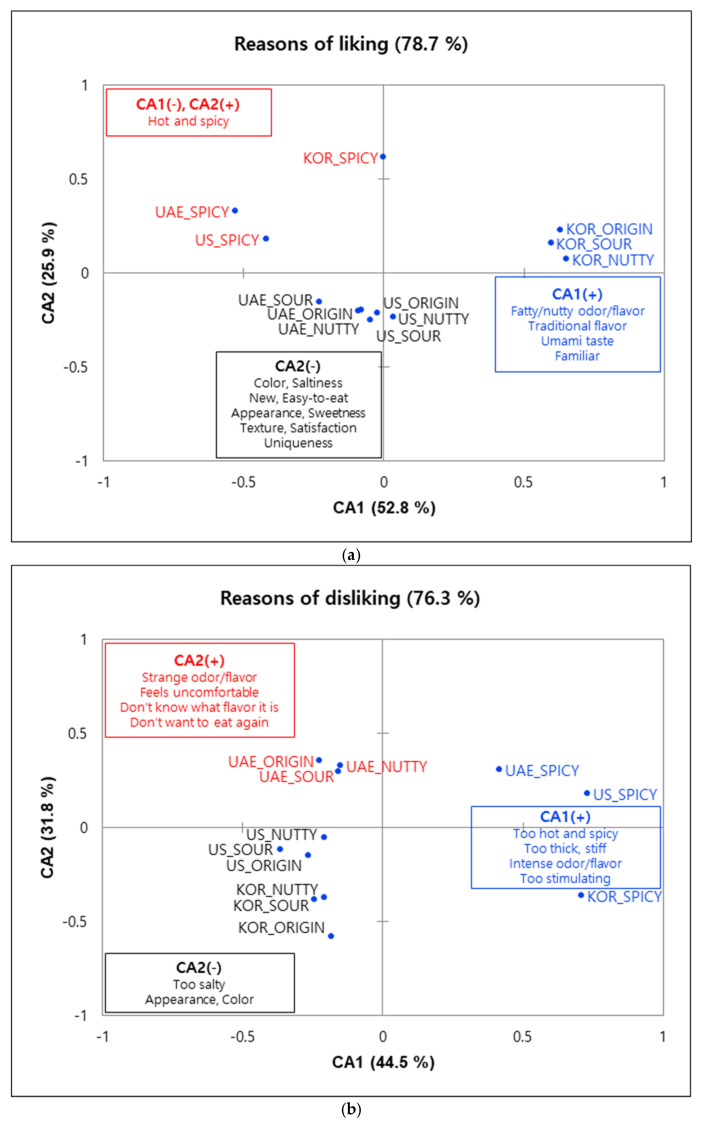
Correspondence analysis (CA) plot of (**a**) reasons for liking attributes and their corresponding salad dressing sample loadings; (**b**) reasons for disliking attributes and their sample loadings. Blue dots indicate the sample loadings evaluated by the consumers in the three countries and attributes in the boxes refer to the check-all-that-apply (CATA) liking and disliking terms.

CA was carried out with CATA terms to summarize the reasons for liking and disliking each of the four samples in the three countries (Figure 2). For the CA of reasons for liking (Figure 2a), the CA1 axis was contrasted between origin, sour, and nutty samples evaluated by Korean subjects with the spicy sample evaluated by UAE and US subjects. Korean subjects liked origin, sour, and nutty samples because of their fatty/nutty odor/flavor (50.5%), traditional flavor (30.4%), umami taste (30.0%), and familiar (24.9%). UAE and US subjects liked the spicy sample for its hot and spiciness (UAE 57.1%, US 61.7%). The CA2 axis was defined positively by the characteristic of the spicy sample perceived by KOR consumers as namely hot and spicy (28.6%) and negatively by attributes the UAE and US consumers associated with the original, sour, and nutty samples. UAE and US consumers commonly selected color (33.3%, 30.6%), saltiness (25.2%, 31.1%), new (37.1%, 27.8%), and easy-to-eat (29.0%, 29.4%) as reasons for liking these samples. In addition, US consumers particularly liked the samples for their appearance (32.8%), sweetness (25.0%), fatty/nutty odor/flavor (41.1%), texture (31.1%), satisfaction (26.1%), and uniqueness (26.7%).

The CA plot of reasons for disliking the salad dressings (Figure 2b) showed that the CA1 axis contrasted the attributes related to spicy samples with those of the mild spicy samples. The positive CA1 axis was mainly defined by disliking the hot and spicy flavor (“too intense”). Additionally, KOR consumers disliked the spicy sample because it elicited a “too intense odor/flavor” (26.4%), “too much thickness” (34.1%), and was “too stimulating” (46.2%). Other attributes that were frequently mentioned by each of the countries were aligned along the CA2 axis. KOR consumers mainly disliked the saltiness (38.7%) of the samples (“too much saltiness”). Consumers in the US and UAE commonly disliked the appearance (26.8%, 25.4%) and color (30.0%, 26.7%) of the samples. Interestingly, UAE consumers chose emotional holistic terms, such as “strange odor/flavor” (27.9%), “feels uncomfortable” (25.0%), “don’t know what flavor it is” (24.3%), and “don’t want to eat again” (31.8%) significantly more than KOR or US consumers.

### 3.2. Dipping Sauce

#### 3.2.1. Effect of Product and Country of Origin on Consumer Acceptance and Perception Ratings

The ANOVA result of the dipping sauce samples (Table 5) revealed several significant differences on the attribute ratings, such as overall liking (*p <* 0.000), smell/odor (*p <* 0.01), taste/flavor (*p <* 0.000), and hot and spicy intensity (*p <* 0.000). The country effect affected the familiarity ratings (*p <* 0.000), and the carrier food type influenced the hot and spicy intensity ratings (*p <* 0.000), but these factors did not significantly influence the liking ratings. 

**Figure 3:**
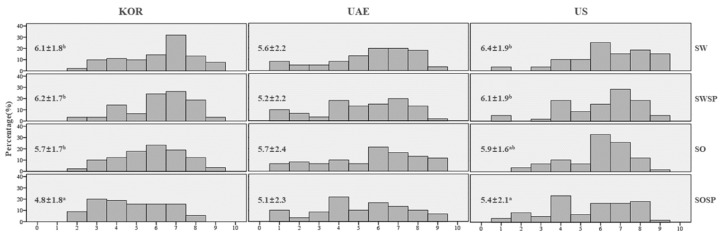
Histogram of overall liking ratings (mean ± SD) of spicy dipping sauce samples evaluated by KOR, UAE, and US consumers. KOR, UAE, US denote South Korean, United Arab Emirates, and Americans, respectively. ^‡^ Samples sharing the same superscript letters within the same country do not differ significantly among the products (α = 0.05). ^§^ SW, SO, SWSP, and SOSP denote sweet, sour, spicy + sweet, and spicy + sour sample, respectively.

The dipping sauce samples were generally more liked (mean overall liking = 5.7) than the salad dressing samples (5.0) across all three populations. Concerning the acceptance levels of hot and spicy dipping sauces, the three countries did not differ significantly in the mean overall liking score. On the contrary, cross-cultural differences were observed between the types of dipping sauce preferred (Figure 3). KOR and US consumers significantly preferred sweet-tasting samples (mean overall liking scores of 6.1–6.2 and 6.1–6.4, respectively) than sour samples (scores of 4.8–5.7 and 5.4–5.9, respectively). By contrast, UAE consumers liked less spicy samples than spicy samples (mean overall liking scores for SW and SO vs. SWSP and SOSP were 5.6 and 5.7 vs. 5.2 and 5.1, respectively).

When exploring the histogram of the overall liking score (Figure 3), KOR consumers displayed a flat distribution for the lowest-rated sample, SOSP (mean score 4.8). UAE consumers as a whole did not show a clear preference. This insignificant sample effect was mainly due to a small mean difference between the samples, as well as relatively large variations observed for individual samples. It is noteworthy that a relatively high proportion of US consumers rated overall liking as “4”, as well as “7” and “8” for spicy samples, regardless of whether it elicited a sweet or sour taste. This observation implies that spicy likers and dislikers coexisted among US consumers.

The samples’ mean attribute intensities and familiarity ratings are listed in Table 6. Consumers in all three countries perceived the hot and spicy intensities of samples to be too high on the JAR scale, but there was cross-cultural disagreement in the perception level of spiciness. KOR consumers perceived sour-tasting samples (SO 5.7, SOSP 6.4) as more intense in heat and spiciness than the sweet-tasting samples (SW 5.1, SWSP 5.1) regardless of actual chili pepper levels. UAE consumers did not significantly differentiate the samples by hot and spicy intensity levels and perceived all samples as significantly too spicy (5.6–6.6). US consumers rated sample SW as closest to the JAR level (5.3) but perceived the other samples as significantly spicier than SW (5.8–6.4). 

In general, dipping sauce samples were more familiar to the consumers than salad dressing samples (familiarity mean values of 4.2 vs. 5.2). Similar to the salad dressing responses, there were large cross-cultural variations in the familiarity ratings of the dipping sauce samples. The samples were more familiar to the KOR consumers (5.9) than the US (5.1) and UAE (4.7) consumers. 

#### 3.2.2. Carrier Food

When evaluating dipping sauces, consumers were asked to freely choose a vegetable stick to use as a carrier food. Cultural differences were observed between the frequently chosen vegetable types. KOR and UAE consumers chose cucumber (65.9%, 63.9%) the most, followed by carrots/baby carrots (16.5%, 18.0%) and broccoli (17.6%, 18.1%). The selection rates of each vegetable type were similar between these two countries, whereas US consumers selected carrots the most (46.7%), followed by cucumber (36.7%) and broccoli (16.7%).

#### 3.2.3. Reasons for Liking and Disliking the Samples

**Figure 4:**
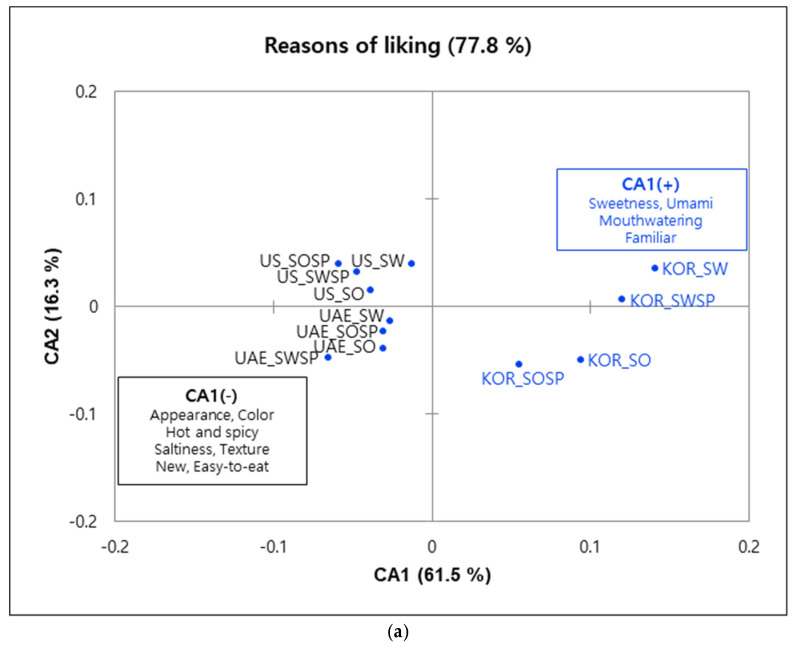

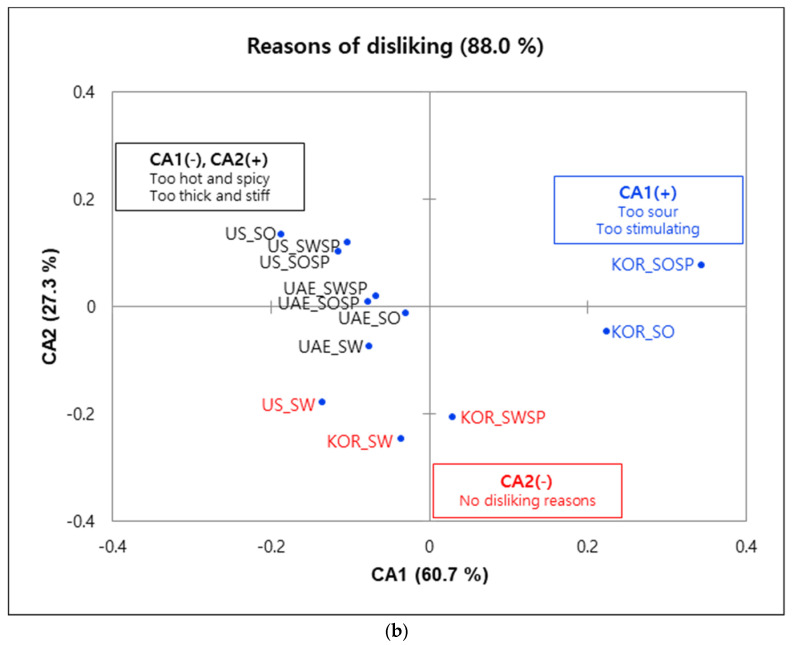
Correspondence analysis plot of (**a**) reasons for liking attributes and corresponding dipping sauce sample loadings; (**b**) reasons for disliking attributes and their sample loadings. Blue dots indicate the sample loadings evaluated by the consumers in the three countries and attributes in the boxes refer to the CATA liking and disliking terms.

Figure 4 shows the first two component plots of CA carried out on the spicy dipping sauce samples. In the reasons for liking, CA1 (61.5%) differentiated the KOR consumers (positive CA1) from the UAE and US consumers (negative CA1). Subsequently, CA 2 contrasted sweet samples (positive direction) vs. sour samples (negative direction) for Korean consumers and additionally contrasted samples evaluated by US vs. UAE consumers, irrespective of the type of samples. Appearance (KOR 33.1%, UAE 42.2%, US 64.6%) and color (KOR 31.9%, UAE 71.7%, US 72.1%) were frequently selected by US and UAE consumers as reasons for liking and showed a strong positive correlation, and were thus loaded on the negative CA1. KOR consumers tended to choose sweet, umami, familiar, and mouthwatering descriptors more frequently than other consumers to describe the liking for the dipping sauce samples. Conversely, hot and spicy, saltiness, new, and easy-to-eat were favorably selected terms among UAE and US consumers. Koreans specifically liked sweet tasting samples because of their sweetness (45.0%), umami (35.0%), familiar (30.0%), mouthwatering (29.4%) while familiar was the only main reason they liked sour samples. 

There were only four CATA terms selected by more than 25% of the consumers as reasons for disliking the dipping sauce samples compared with 12 descriptors for the salad dressing samples. Consumers also chose “no disliking reasons” at a higher rate than observed for the salad dressings, indicating that the salad dressings were less liked than the dipping sauces. CA1 separated the sour-tasting samples disliked by KOR consumers (positive direction) from three of the four samples disliked by US consumers (negative direction). KOR consumers disliked samples SO and SOSP because these sauces were perceived as “too sour” (34.4%) and “too stimulating” (32.2%). US consumers selected “too hot and spicy” (36.7%) and “too thick and stiff” (20.6%) as reasons for disliking samples SO, SWSP, and SOSP. The second component (CA2) roughly differentiated sweet-tasting samples (negative direction) from sour-tasting samples (positive direction). No disliking reasons, which carries the meaning of no reason to dislike, were strongly and positively associated with the sweet-tasting samples.

## 4. Discussion

The present study investigated the cross-cultural consumer acceptances of salad dressings and dipping sauces made from Korean fermented seasonings. KOR, UAE, and US consumers generally liked dipping sauces eliciting chili spiciness more than salad dressing having a distinct fermented flavor. Protein or carbohydrate-based fermented flavors are difficult to be accepted when the flavor is culturally unfamiliar [34,35,36]. UAE consumers showed the lowest scores for flavor familiarity and mean overall liking of the salad dressing samples among the consumers from the three countries. The unfamiliar fermented flavor of the dressing samples may be responsible for driving the dislike of salad dressings by UAE consumers.

Concerning the preferred flavor types of the salad dressing samples, KOR consumers preferred nutty-flavored samples. Nutty flavor (translated as “goso”) has been reported as one of the most notable and positive flavors appreciated by KOR consumers in Korean cuisines [37,38]. This cultural preference for nuttiness seemed to be reflected in the liking for salad dressing with a nutty flavor among KOR consumers. Conversely, a spicy note in the sample elicited a positive response among US and UAE consumers. This result may be in line with the growing popularity of spiciness in ethnic dishes globally [16,39,40]. Additionally, the spicy note in the dressing may have masked the negative fermented flavor in the sample since spiciness has been reported to suppress particular odors, tastes, and flavors [41,42,43]. KOR consumers considered sensory and nonsensory attributes, such as fatty/nutty odor/flavor, traditional flavor, umami taste, and familiarity, as important positive attributes. However, UAE and US consumers considered color, saltiness, new, and easy-to-eat as important positive drivers. KOR consumers perceived spiciness in the dressing samples as “too stimulating”, and this factor acted as a negative driver of liking. In UAE consumers who rated the liking score of the salad dressing samples the lowest, the reasons for disliking the samples were “strange odor/flavor”, “feel uncomfortable”, and other negative holistic or emotional terms. Similar to the results of previous studies [12,44], familiarity contributed to the liking ratings of the samples because either the sample with the highest-rated overall liking was also rated the highest in familiarity (KOR consumers) or the sample with the lowest-rated overall liking was also rated the lowest in familiarity (US consumers).

As mentioned above, the dipping sauce samples were generally more liked than the salad dressing samples. There are two possible reasons for this outcome. First, the general liking of a spicy flavor among most of the consumers may have resulted in the relatively higher acceptance ratings for dipping sauce. Our finding corroborates the reports stating that the chili spiciness is widely accepted, and the consumption rate of chili peppers has increased globally [39]. The second reason could be that by allowing consumers to choose a vegetable to use as a carrier food for the dipping sauce but not for the salad dressing tasting, it may have induced positive attitudes toward the overall dipping sauce evaluation [45,46].

Regarding the preferred flavor of dipping sauces, KOR and US consumers significantly liked sweet rather than sour-tasting samples. By contrast, UAE consumers showed tendencies of liking less spicy samples than spicy samples irrespective of whether the sample elicited sourness or sweetness in addition to spiciness. KOR consumers tended to choose sweet, umami, familiar, and mouthwatering descriptors at a higher rate than the US and UAE consumers. Hot and spicy, saltiness, new, and easy-to-eat were favorably selected terms among UAE and US consumers. Most consumers, regardless of their country of origin, tended to dislike samples when they perceived the taste to be “too stimulating” to the senses or “too spicy”.

One of the interesting findings in the present study was the cultural difference in the liking response to the spiciness between the two food types. The presence of spiciness in the salad dressings was positive for UAE and US consumers only. On the contrary, intense spiciness in dipping sauce was a negative driver for UAE consumers, and spiciness intensity was not a critical factor for US and KOR consumers when spiciness was the dominant flavor property. Among the various sensory attributes, spiciness shows the widest variation in individual preference for its presence or intensity level in foods [47,48,49]. This diversity in preference for chili spiciness is due to factors such as diversity in cultural exposure to the flavor, genetic influence, gender, and personal traits. Chili spiciness has been traditionally enjoyed by countries like Mexico, Indonesia, Mainland China, and Korea [50]. Its popularity in other parts of the world, such as Europe, Middle Eastern Asia, and North America, is relatively recent. It has been observed that the chili pungency can be perceived as aversive when the consumers are not frequently exposed to the stimulus [51,52,53], but that pleasure is learned when the exposure to the stimuli is repeated. Additionally, several studies have reported that frequent chili consumers perceive the burning/spicy intensity of chili as less intense than less frequent chili consumers [54,55,56] and this was partly shown in the present study. That is, those who are not used to spiciness perceive the spiciness to be too intense (i.e., spicy intensity ratings in UAE consumers) compared with those who are exposed to the stimulus frequently (KOR consumers).

As indicated from the results of Levene’s test for equality of variances, which showed significant differences among the three countries, the distribution of overall liking scores within each country provided useful information on understanding the acceptance of samples. Particularly, a bipolar distribution of overall liking ratings was observed in the liking of nutty salad dressing and spicy (SWSP, SOSP) dipping sauce samples evaluated by US consumers. These bipolar distributions imply that consumers had heterogeneous preferences.

## 5. Conclusions

To summarize and conclude the findings of the study, KOR consumers liked the nutty flavor, whereas UAE consumers preferred the spicy flavor, and US consumers showed comparable levels of liking for spicy, sour, and nutty flavors among the four *doenjang*-based dressing samples. Consumers in all three countries generally liked dipping sauces more than salad dressings. Both KOR and US consumers liked spicy dipping sauces eliciting a sweet taste. Although UAE consumers did not significantly differentiate the four spicy dipping sauces by liking score, they tended to prefer less spicy samples over spicier samples. Overall, the presence of spiciness and the level of spiciness present in the salad dressings and the spicy dipping sauces were the most critical factors affecting the liking for the tested samples. The acceptances for the spicy-eliciting samples varied with the sauce type, the consumer’s country of origin, and the consumer’s general preference for spiciness. Thus, the optimal spicy level for these types of ethnic salad dressings or dipping sauces has to be carefully verified and tuned to the cultural-culinary backgrounds of the target consumers.

The current study carries several limitations in generalizing the results to a larger group of population due to the following reasons. Consumer subjects in the three countries had a relatively narrow range of age groups and the age group of US consumers was older than that of Korea and UAE consumers. Additionally, the ratio of female and male was unequal. Although the four samples within the salad dressing or dipping sauce experiments were counterbalanced in terms of serving order, salad dressing samples were tasted prior to the dipping sauce samples. Thus, order effect may have been present and somewhat influenced the evaluation of the dipping sauces.

The effect of food neophobic traits of consumers, which has shown to be a significant factor in delineating the liking for a food product [57], was not particularly investigated in the present study due to the limited numbers of consumer subjects. Further investigations on the relationships between individual’s psychographic attitudes, such as food neophobic trait and the liking of ethnic foods in cross-cultural contexts, may offer a useful insight in developing marketing strategies for these food items in different countries.

## Figures and Tables

**Table 1:** Consumers’ demographic information.

Group	Category	KOR ^†^	UAE	US
Number of Subjects		91	70	60
Age	Average ± S.D.	27.6 ± 10.0	23.4 ± 8.8	39.4 ± 13.5
Sex	Male	30 (33%)	9 (12.9%)	27 (45%)
	Female	61 (67%)	44 (62.9%)	33 (55%)
	No response	-	17 (24.2%)	-
Ethnicity	Caucasian	-	1 (1.4%)	34 (56.7%)
	African American	-	1 (1.4%)	8 (13.3%)
	Arab	-	51 (72.9%)	-
	Asian	91 (100%)	2 (2.9%)	18 (30%)
	Multi-racial	-	2 (2.9%)	-
	No response	-	13 (18.6%)	

^†^ KOR, UAE, US denote South Korean, United Arab Emirates, and Americans, respectively.

**Table 2:** Products’ ingredient information.

Type	Sample	Main Ingredients
Salad dressing	Original	Conventional-type *doenjang* (soybean, wheat flour, wheat grain, salt, flavor enhancers IMP and GMP ^†^)
	Sour	Original + Apple vinegar
	Nutty	Original + Peanut paste
	Spicy	Original + Chili pepper powder
Spicy dipping sauce	Sweet	Wheat grain, wheat, starch syrup, high-fructose corn syrup, tomato ketchup, onion, garlic
	Sour	Wheat grain, wheat, high-fructose corn syrup, vinegar, citric acid
	Spicy + Sweet	Sweet sample + Chili pepper powder
	Spicy + Sour	Sour sample + Chili pepper powder

^†^ IMP and GMP denote inosine monophosphate and guanosine monophosphate, respectively.

**Table 3:** Influence of sample, country, and sample × country interaction effects on the ratings of liking, intensity, and familiarity of salad dressing samples.

Source of Variation	Sample	Country	Sample × Country
Overall liking	0.002 ^†^	0.000	0.000
Appearance liking	0.002	0.000	0.000
Smell/Odor liking	0.001	0.000	0.000
Taste/Flavor liking	0.003	0.000	0.000
Texture liking	0.514	0.000	0.000
Saltiness intensity	0.152	0.000	0.002
Thickness/viscosity intensity	0.000	0.055	0.000
Familiarity	0.210	0.000	0.001

^†^ Value indicates *p*-value of each factor, obtained from ANOVA analyzed by general linear model, on each attribute. The significance level α is 0.05.

**Table 4:** Ratings (mean ± SD) of intensities and familiarity of salad dressing samples evaluated by the KOR, UAE, and US ^†^ consumers.

Country	Sample	Saltiness	Thickness/Viscosity	Familiarity
KOR	Original	6.8 ± 1.4 ^b,^^‡,^^*,^^§^	5.1 ± 1.1 ^b^	5.5 ± 1.8 ^b,c^
	Sour	6.5 ± 1.4 ^a,*^	4.1 ± 1.3 ^a,*^	5.1 ± 1.8 ^a,b^
	Nutty	6.3 ± 1.3 ^a,*^	5.7 ± 1.3 ^c,*^	5.7 ± 1.8 ^c^
	Spicy	6.9 ± 1.3 ^b,*^	6.4 ± 1.6 ^d,*^	4.9 ± 2.2 ^a^
UAE	Original	5 ± 2.2	5.3 ± 1.9 ^a,b^	3.1 ± 2.2 ^a^
	Sour	4.8 ± 2.1	4.8 ± 2.1 ^a^	3.1 ± 2.1 ^a^
	Nutty	5.2 ± 2.1	5.7 ± 2.2 ^b,c,*^	3.7 ± 2.2 ^b^
	Spicy	4.9 ± 2.1	6 ± 1.9 ^c, *^	3.8 ± 2.2 ^b^
US	Original	6.1 ± 2.2 ^b,*^	6.2 ± 1.5 ^b,*^	3.7 ± 2.4
	Sour	5.9 ± 1.7 ^b,*^	4.9 ± 1.5 ^a^	4.1 ± 2.4
	Nutty	6.0 ± 1.8 ^b,*^	6.2 ± 1.6^b *^	4 ± 2.4
	Spicy	5.3 ± 1.7 ^a^	5.8 ± 1.4^b *^	3.9 ± 2.3

^†^ KOR, UAE, US denote South Korean, United Arab Emirates, and Americans, respectively. ^‡^ Samples sharing the same superscript letters within the same country do not differ significantly among the products (α = 0.05). *^§^ Significantly different from value “5 = just-about-right” analyzed by the one-sample *t*-test method.

**Table 5:** Influence of sample, country, carrier food main effects, and their interaction effects on the ratings of liking, intensity, and familiarity of dipping sauce samples.

Source of Variation	Sample	Country	Carrier	Sample × Country	Sample × Carrier	Country × Carrier	Sample × Country × Carrier
Overall liking	0.000 ^†^	0.691	0.282	0.453	0.856	0.017	0.456
Appearance	0.163	0.151	0.528	0.215	0.664	0.182	0.355
Smell/Odor	0.005	0.914	0.230	0.510	0.458	0.192	0.873
Taste/Flavor	0.000	0.792	0.341	0.273	0.836	0.055	0.793
Texture	0.052	0.314	0.211	0.229	0.752	0.315	0.481
Hot and spicy intensity	0.000	0.648	0.000	0.004	0.818	0.457	0.121
Thickness/Viscosity intensity	0.059	0.063	0.164	0.000	0.007	0.431	0.056
Familiarity	0.935	0.000	0.658	0.075	0.554	0.684	0.843

^†^ Value indicates *p*-value of each factor, obtained from ANOVA analyzed by general linear model, on each attribute. The significance level α is 0.05.

**Table 6:** Ratings (mean ± SD) of intensities and familiarity of spicy dipping sauce samples evaluated by the KOR, UAE, and US ^†^ consumers.

Country	Sample	Hot and Spicy	Thickness	Familiarity
KOR	SW	5.1 ± 1.3 ^a,^^‡,^^*,^^§^	5.5 ± 1.0,^b,*^	5.8 ± 1.7 ^a^
	SWSP	5.1 ± 1.2 ^a^	5.3 ± 0.8 ^b,*^	5.9 ± 1.7 ^a,b^
	SO	5.7 ± 1.4 ^b,*^	5.3 ± 1.0 ^b,*^	6.3 ± 1.7 ^b^
	SOSP	6.4 ± 1.4 ^c,*^	4.9 ± 0.9 ^a^	5.8 ± 2.1 ^a^
UAE	SW	5.6 ± 2.1 ^*^	5.8 ± 1.9 ^*^	4.7 ± 2.2
	SWSP	6.5 ± 2.3 ^*^	5.5 ± 1.7 ^*^	4.4 ± 2.2
	SO	6.1 ± 2.0 ^*^	5.5 ± 1.9	4.8 ± 2.3
	SOSP	6.6 ± 2.2 ^*^	5.9 ± 1.8 ^*^	5 ± 2.4
US	SW	5.3 ± 1.7 ^a^	5.2 ± 1.3 ^a^	4.4 ± 2.2
	SWSP	6.4 ± 1.8 ^b,*^	5.2 ± 1.3 ^a^	4.3 ± 2.2
	SO	5.8 ± 1.9 ^a,b,*^	6.3 ± 1.7 ^b,*^	3.9 ± 2.1
	SOSP	6.2 ± 2.1 ^b,*^	5.8 ± 1.6 ^b,*^	4.2 ± 2.2

^†^ KOR, UAE, US denote South Korean, United Arab Emirates, and Americans, respectively. ^‡^ Samples sharing the same superscript letters within the same country do not differ significantly among the products (α = 0.05). *^,§^ Significantly different from value “5 = just-about-right” analyzed by the one-sample *t*-test method.

## References

[1] Sasaki M., Nunomura N., Caballero B., Trugo L., Finglas P.M. (2007). Fermented foods/soy(soya) sauce. Encyclopedia of Food Sciences and Nutrition.

[2] Hong J.H., Park H.S., Chung S.J., Chung L., Cha S.M., Lê S., Kim K.O. (2014). Effect of familiarity on a cross-cultural acceptance of a sweet ethnic food: A case study with Korean traditional cookie (Yackwa). J. Sens. Stud..

[3] Burgess P.J. (2014). Modification of a traditional Korean food product (*Gochujang*) to enhance its consumer acceptability as an ethnic food. J. Ethn. Foods.

[4] Camarena D.M., Sanjuán A.I., Philippidis G. (2011). Influence of ethnocentrism and neo-phobia on ethnic food consumption in Spain. Appetite.

[5] Verbeke W., López G.P. (2005). Ethnic food attitudes and behaviour among Belgians and Hispanics living in Belgium. Brit. Food J..

[6] Bell B., Adhikari K., Chambers E., Cherdchu P., Suwonsichon T. (2011). Ethnic food awareness and perceptions of consumers in Thailand and the United States. Food Sci. Nutr..

[7] Chung L., Chung S.J., Kim J.Y., Kim K.O., O’Mahony M., Vickers Z., Cha S.M., Ishii R., Baures K., Kim H.R. (2012). Comparing the liking for Korean style salad dressings and beverages between US and Korean consumers: Effects of sensory and non-sensory factors. Food Qual. Prefer..

[8] Hong J.H., Lee K.W., Chung S.J., Chung L., Kim H.R., Kim K.O. (2012). Sensory characteristics and cross-cultural comparisons of consumer acceptability for *Gochujang* dressing. Food Sci. Biotechnol..

[9] López-Guzmán T., Sánchez-Cañizares S. (2012). Culinary tourism in Córdoba (Spain). Br. Food J..

[10] Ottenbacher M.C., Harrington R.J. (2013). A case study of a culinary tourism campaign in Germany: Implications for strategy making and successful implementation. J. Hosp. Tour. Res..

[11] Wijaya S. (2019). Indonesian food culture mapping: A starter contribution to promote Indonesian culinary tourism. J. Ethn. Food.

[12] Grand View Research (2018). Sauces, Dressings & Condiments Market Size, Share & Trends Analysis Report by Product (Cooking Sauces, Dips), by Distribution Channel, by Region, and Segment Forecasts, 2019—2025. https://www.grandviewresearch.com/industry-analysis/sauces-dressings-condiments-market.

[13] Kim S., Choe J., Lee A. (2016). Efforts to globalize a national food: Market segmentation by reasons for ethnic food preferences. Int. J. Contemp. Hosp. Manag..

[14] Park S., Jang H. (2017). Strategies to Expand Export in Halal Market: Focusing on the Cases of Small and Medium Enterprises.

[15] Im P., Han J.H., Kim Y.C., Lee B., Kim M.Y., Chang Y., Yu S., Lee Y. (2015). Effects of guar gum on quality of soft tofu stew sauce. J. Korean Soc. Food Sci. Nutr..

[16] Kim H.J., Chung S.J., Kim K.O., Nielsen B., Ishii R., O’Mahony M. (2018). A cross-cultural study of acceptability and food pairing for hot sauces. Appetite.

[17] Hong J.H., Yoon E.K., Chung S.J., Chung L., Cha S.M., O’mahony M., Vickers Z., Kim K.O. (2011). Sensory characteristics and cross-cultural consumer acceptability of Bulgogi (Korean traditional barbecued beef). J. Food Sci..

[18] Jo S.G., Lee S.M., Sohn K.H., Kim K.O. (2015). Sensory characteristics and cross-cultural acceptability of Chinese and Korean consumers for ready-to-heat (RTH) type bulgogi (Korean traditional barbecued beef). Food Sci. Biotechnol..

[19] Kang N.E., Jo S.K., Lee S.M., Kim K.O. (2014). Cross-cultural investigation on Chinese and Korean consumers’ reasons for liking and disliking for bulgogi using check-all-that-apply questionnaire. J. Korean Soc. Food Cult..

[20] Choi J.H., Gwak M.J., Chung S.J., Kim K.O., O’Mahony M., Ishii R., Bae Y.W. (2015). Identifying the drivers of liking by investigating the reasons for (dis) liking using CATA in cross-cultural context: A case study on barbecue sauce. J. Sci. Food Agric..

[21] Moskowitz H.W., Kumaraiah V., Sharma K.N., Jacobs H.L., Sharma S.D. (1975). Cross-cultural differences in simple taste preferences. Science.

[22] Druz L.L., Baldwin R.E. (1982). Taste thresholds and hedonic responses of panels representing three nationalities. J. Food Sci..

[23] Prescott J., Young O., Zhang S., Cummings T. (2004). Effects of added “flavour principles” on liking and familiarity of a sheepmeat product: A comparison of Singaporean and New Zealand consumers. Food Qual. Prefer..

[24] Dolgopolova I., Teuber R., Bruschi V. (2015). Consumers’ perceptions of functional foods: Trust and food-neophobia in a cross-cultural context. Int. J. Consum. Stud..

[25] Jang S.H., Kim M.J., Lim J., Hong J.H. (2016). Cross-cultural comparison of consumer acceptability of kimchi with different degree of fermentation. J. Sens. Stud..

[26] Mialon V.S., Clark M.R., Leppard P.I., Cox D.N. (2002). The effect of dietary fibre information on consumer responses to breads and “English” muffins: A cross-cultural study. Food Qual. Prefer..

[27] Varela P., Ares G. (2012). Sensory profiling, the blurred line between sensory and consumer science. A review of novel methods for product characterization. Food Res. Int..

[28] Rasinski K.A., Mingay D., Bradburn N.M. (1994). Do respondents really mark all that apply on self-administered questions?. Public Opin. Quart..

[29] Adams J., Williams A., Lancaster B., Foley M. Advantages and uses of check-all-that-apply response compared to traditional scaling of attributes for salty snacks. Proceedings of the 7th Pangborn Sensory Science Symposium.

[30] Gunaratne T.M., Viejo C.G., Fuentes S., Torrico D.D., Gunaratne N.M., Ashman H., Dunshea F.R. (2019). Development of emotion lexicons to describe chocolate using the Check-All-That-Apply (CATA) methodology across Asian and Western groups. Food Res. Int..

[31] Jaeger S.R., Chheang S.L., Jin Y., Bava C.M., Gimenez A., Vidal L. (2013). Check-all-that-apply (CATA) responses elicited by consumers: Within-assessor reproducibility and stability of sensory product characterizations. Food Qual. Prefer..

[32] Peryam D.R., Pilgrim F.J. (1957). Hedonic scale method of measuring food preferences. Food Technol..

[33] Pitts S., Rotham L., Parker M.J. (2009). Mean versus scale mid-point, Appendix, F. Just-About-Right (JAR) Scales: Design, Usage, Benefits and Risks.

[34] Zhang X.Y., Guo H.Y., Zhao L., Sun W.F., Zeng S.S., Lu X.M., Cao X., Ren F.Z. (2011). Sensory profile and Beijing youth preference of seven cheese varieties. Food Qual. Prefer..

[35] Akissoé N.H., Sacca C., Declemy A.L., Bechoff A., Anihouvi V.B., Dalodé G., Pallet D., Fliedel G., Mestres C., Hounhouigan J.D. (2015). Cross-cultural acceptance of a traditional yoghurt-like product made from fermented cereal. J. Sci. Food Agric..

[36] Go J.E., Kim M.R., Chung S.J. (2017). Acquired (dis)liking of natural cheese in different repeated exposure environment. Food Res. Int..

[37] Hwang S.H., Hong J.H. (2013). Sensory drivers of goso flavour in soymilk: Understanding a complex traditional sensory attribute. Food Qual Prefer..

[38] Kim S.H., Petard N., Hong J.H. (2018). What is lost in translation: A cross-cultural study to compare the concept of nuttiness and its perception in soymilk among Korean, Chinese, and Western groups. Food Res. Int..

[39] Spence C. (2018). Why is piquant/spicy food so popular?. Int. J. Gastron. Food Sci..

[40] Park H.-J., Ko J.-M., Lim J., Hong J.-H. (2020). American consumers’ perception and acceptance of an ethnic food with strong flavor: A case study of Kimchi with varying levels of red pepper and fish sauce. J. Sci. Food Agric..

[41] Lawless H., Rozin P., Shenker J. (1985). Effects of oral capsaicin on gustatory, olfactory and irritant sensations and flavor identification in humans who regularly or rarely consume chili pepper. Chem. Senses.

[42] Reinbach H.C., Meinert L., Ballabio D., Aaslyng M.D., Bredie W.L.P., Olsena K., Møller P. (2007). Interactions between oral burn, meat flavor and texture in chili spiced pork patties evaluated by time-intensity. Food Qual. Prefer..

[43] Kapaun C.L., Dando R. (2017). Deconvoluting physical and chemical heat: Temperature and spiciness influence flavor differently. Physiol. Behav..

[44] Torrico D.D., Fuentes S., Viejo C.G., Ashman H., Dunshea F.R. (2019). Cross-cultural effects of food product familiarity on sensory acceptability and non-invasive physiological responses of consumers. Food Res. Int..

[45] De Graaf C., Cardello A.V., Kramer F.M., Lesher L.L., Meiselman H.L., Schutz H.G. (2005). A comparison between liking ratings obtained under laboratory and field conditions: The role of choice. Appetite.

[46] Chung S.J., Vickers Z. (2007). Long-term acceptability and choice of teas differing in sweetness. Food Qual. Prefer..

[47] Spinelli S., De Toffoli A., Dinnella C., Laureati M., Pagliarini E., Bendini A. (2018). Personality traits and gender influence liking and choice of food pungency. Food Qual. Prefer..

[48] Trachootham D., Sato-Kuriwada S., Lam-Ubol A., Promkam C., Chotechuang N., Sasano T., Shoji N. (2017). Differences in taste perception and spicy preference: A Thai—Japanese cross-cultural study. Chem. Senses.

[49] Scott N.O., Burgess B., Tepper B.J. (2019). Perception and liking of soups flavored with chipotle chili and ginger extracts: Effects of PROP taster status, personality traits and emotions. Food Qual. Prefer..

[50] Rozin E. (1983). Ethnic Cuisine: The Flavor-Principle Cookbook.

[51] Rozin P., Schiller D. (1980). The nature and acquisition of a preference for chili pepper by humans. Motiv. Emot..

[52] Rozin P., Green B.G., Mason J.R., Kare M.R. (1990). Getting to like the burn of chili pepper. Biological, psychological and cultural perspectives. Chemical Senses, Irritation.

[53] Carstens E., Carstens M.I., Dessirier J.M., O’Mahony M., Simons C.T., Sudo M., Sudo S. (2002). It hurts so good: Oral irritation by spices and carbonated drinks and the underlying neural mechanisms. Food Qual. Prefer..

[54] Cowart B.J. (1987). Oral chemical irritation: Does it reduce perceived taste intensity?. Chem. Senses.

[55] Stevenson R.J., Prescott J. (1994). The effects of prior experience with capsaicin on ratings of its burn. Chem. Senses.

[56] Nolden A.A., Hayes J.E. (2017). Perceptual and affective responses to sampled capsaicin differ by reported intake. Food Qual. Prefer..

[57] Pliner P., Hobden K. (1992). Development of a scale to measure the trait of food neophobia in humans. Appetite.

